# External load profile during different sport-specific activities in semi-professional soccer players

**DOI:** 10.1186/s13102-023-00633-3

**Published:** 2023-02-22

**Authors:** Guglielmo Pillitteri, Valerio Giustino, Marco Petrucci, Alessio Rossi, Marianna Bellafiore, Ewan Thomas, Angelo Iovane, Antonino Bianco, Antonio Palma, Giuseppe Battaglia

**Affiliations:** 1 Sport and Exercise Sciences Research Unit, Department of Psychology, Educational Science and Human Movement, University of Palermo, Palermo, Italy; 2 PhD Program in Health Promotion and Cognitive Sciences, University of Palermo, Palermo, Italy; 3 Palermo, FC Italy; 4 Department of Computer Science, University of Pisa, Pisa, Italy; 5 Institute of Information Science and Technologies (ISTI), National Research Council (CNR), Pisa, Italy

**Keywords:** Training load, GPS, Physical demand, SSG, Playing position

## Abstract

**Background:**

Global Positioning System (GPS) devices are widely used in soccer for monitoring external load (EL) indicators with the aim of maximizing sports performance.The aim of this study was to investigate the EL indicators differences in players of different playing positions (i.e., central backs, external strikers, fullbacks, midfielders, strikers, wide midfielder) between and within different sport-specific tasks and official matches.

**Methods:**

1932 observations from 28 semi-professional soccer players (age: 25 ± 6 years, height: 183 ± 6 cm, weight: 75.2 ± 7 kg) were collected through GPS devices (Qstarz BT-Q1000EX, 10 Hz) during the season 2019–2020. Participants were monitored during Official Match (OM), Friendly Matches (FM), Small Sided Games (SSG), and Match-Based Exercises (MBE). Metabolic (i.e., metabolic power, percentage of metabolic power > 35w, number of intense actions per minute*,* distance per minute, passive recovery time per minute) and neuromuscular indicators (i.e., percentage of intense accelerations, percentage of intense decelerations, change of direction per min > 30°) were recorded during each task.

**Results:**

Statistically significant differences were detected in EL indicators between playing positions within each task and between tasks. In particular, results from the two-way ANOVA tests showed significant interaction, but with small effect size, in all the EL indicators between playing positions for each task and within tasks. Moreover, statistical differences, but with small effect size, between playing positions were detected in each task and for each EL indicator. Finally, the strongest statistical differences (with large effect size) were detected between tasks for each EL indicator. Details of the Tukey post-hoc analysis reporting the pairwise comparisons within and between tasks with playing positions are also provided.

**Conclusion:**

In semi-professional soccer players, different metabolic and neuromuscular performance were detected in different playing position between and within different tasks and official matches. Coaches should consider the different physical responses related to different physical tasks and playing position to design the most appropriate training program.

## Introduction

Soccer is an open skills team sport in which players are interconnected in a complex system characterized by technical-tactical components that are supported by physical and physiological factors [1]. Soccer performance is described by unpredictable intermittent cyclic and acyclic activities characterized by role-specific physical demands [2–4]. Understanding the physical demands required during soccer matches for specific playing position and more in details for each task is important for coaches in order to appropriately schedule the training program with the aim of maximizing the effect of training while minimizing the risk of injury.

To improve soccer players’ performance while reducing injury risk, coaches should assess training and match loads in relation to playing position on the field to optimize training planning [3, 13–21]. Specifically, EL is usually described by the total distance, range of speed covered, accelerations, metabolic power [9], and other derived measures. Global Positioning System (GPS) technology has been largely used by team sport coaches to assess EL allowing them to make time-motion analysis of on-field performance during technical-tactical tasks and official matches [16, 22, 23]. In this way, GPS was used to detect relevant components of player movement patterns including derived measures of speed, distance, acceleration, and metabolic power [9, 24].

The main specific tasks proposed by coaches during soccer training include Small Sided Games (SSG), match-based exercises (MBE) and friendly matches (FM). Coaches could schedule different tasks in order to provide at their players different EL stimuli inducing different individuals’ adaptations. In fact, different ELs induce specific internal load (IL) responses because different sports specific task induces different psychological, physiological, biochemical, metabolic and biomechanical stress stimuli [25, 26]. Actually, different external and internal load responses are shown considering different typology of soccer training task [27, 28]. SSGs represent tasks with specific technical-tactical rules performed in small spaces of the pitch with a reduced number of players compared to regular matches. These tasks elicit physical, tactical, psychological and techniques components [29–35]. Differently MBE represents tasks more similar to regular matches which are organized in larger spaces and with a larger number of players than SSG. Moreover, ~ 300 m^2^ is the theoretical match reference density of a regular match [36, 37] while soccer-specific tasks are performed using ~ 100 m^2^ or ~ 200 m^2^ per player [37–39]. Due to these differences, various intensities are detected in each of them [22, 40]. Player’s density on the pitch (i.e., area per player, m^2^/player) represent a key factors of the SSG that determines different external load responses and estimates physiological match demands in elite soccer players [41]. Indeed, Riboli et al., found that to reach higher thresholds at high speed, a higher density is required regardless of the type of SSG [41, 42]. Moreover, larger area per player determines higher total distance, high and very high-speed distance and sprint. It worth noting that a minimum of ~ 200 m^2^/player could properly stimulate the high speed and sprint activities in youth players [28]. Authors observed that both EL and IL can be affected by some variables such as game rules, objectives, number of players per team, pitch size and coaches’ verbal stimulation [35, 42–44]. Some studies focused on accelerations and decelerations involving both metabolic and neuromuscular systems and described the physiological load requests in SSG [3, 35, 41, 42, 45, 46]. These studies showed that in SSGs with low density results in high number of accelerations, decelerations and changes of direction. Differently, in larger spaces, due to the high-speed running achieved by players, high metabolic components were detected [36, 41, 42]. Moreover, higher peak speeds and very high speeds were detected during official and friendly matches compared to SSG [10, 46, 47]. Interesting, one study found that SSG with goalkeeper requires higher density compared to SSG without goalkeeper. Specifically, authors reported that a density of 350 m^2^/player should be used to recreate the 4-minPeak match-play demands for high-speed to very high-speed activities using SSG with goalkeeper [42]. These results could be due to greater tactical components as the presence of goalkeeper requires great density to increase the intensity of the activities [41]. Hence, the SSG tasks showed high-intensity activity that involves neuromuscular factors highlighting the important role of acceleration. In this way some EL differences were detected in SSG compared to matches [46, 48].

It is worth noting that physical demand is highly related to playing positions on the pitch since roles have specific technical-tactical requests strictly related to different physical, physiological, energetic and biomechanical components [2, 21, 42, 49]. For example, the longest distance covered at high intensity is achieved by wide midfielders and fullbacks [14, 50]. Additionally, the use of SSG with goalkeeper results in performance differences between positions, while this result was not found in SSG without goalkeeper [41, 42]. However, soccer players’ performance was found to be strictly related to playing position [2–5, 42, 51–56].

To the best of our knowledge, although there are many studies that have investigated differences in EL indicators between and within official matches and soccer-specific tasks considering soccer playing position [26, 57–59], the novelty of our study was to include several soccer-specific tasks for both SSG and MBE and to recruit semi-professional soccer players.. Understanding EL response between tasks and official matches (OM) and within tasks for each different playing position could be necessary to better schedule training macrocycle helping practitioners to modulate appropriately training intensity and target. We hypothesize differences in EL indicators for each playing position within and between tasks and official matches. Hence, the aim of this study was to investigate differences in EL indicators, specifically strength and metabolic indicators, between and within OM, FM, SSG, and MBE in different playing positions of semi-professional soccer players.

## Methods

### Experimental approach to the problem

In this cross-sectional study, players from a semi-professional Italian soccer club were monitored using a GPS device during OM, FM, and sport-specific tasks (i.e., SSG and MBE) during an entire season (2019–2020).

### Participants

Twenty-eight semi-professional soccer players (age: 25 ± 6 years, height: 183 ± 6 cm, weight: 75.2 ± 7 kg) competing in the fourth Italian (Serie D) division were analysed. The following inclusion criteria were considered: (1) semi-professional male soccer players; (2) no injury in the previous six months. Based on the exclusion criteria, only the goalkeepers were not eligible for the study. The duration of the training sessions was approximately 100 min and mainly included technical-tactical team tasks along with some general physical exercise without ball (e.g., high intensity interval training, HIIT) [60, 61]. Strength and conditioning exercises were performed twice a week (i.e., one session in the gym and one session on the pitch). All participants signed an informed consent before taking part to the data collection. The study, which complies with the principles of the Declaration of Helsinki, was approved by the Bioethics Committee of the University of Palermo (n. 68/2021).

### Procedures

Participants were monitored during OM and the following three soccer-specific tasks: FM, SSG, and MBE. The choice to compare OM and FM, SSG and MBE was based on the fact that these tasks are performed in a field with a similar proportion of the official one and with similar players’ density, but with fixed rules. In particular, we compared the usual theoretical match density (both for OM and FM) referred to as ~ 300 m^2^ with the different densities according to m^2^/player of soccer-specific tasks [37–39]. SSG and MBE have been included considering a range from 62 to 176 m^2^/player (~ 100 m^2^/player) and from 178 to 260 m^2^/player (~ 200 m^2^/player) for SSG and MBE, respectively [37, 38]. Both matches and soccer-specific tasks were played on a third-generation artificial pitch or natural grass. All players performed a 15-min warm-up before performing MBE and SSG and a typical 25-min pre-match warm-up before OM and FM.

Data was collected through a GPS unit (Qstarz BT-Q1000EX, 10 Hz) [62, 63] positioned on the upper back and inserted in a special vest. The GPS device was started 15 min prior to the assessment to make available the acquisition of satellite signals. After fixing the GPS, the average number of satellites in view was 12, and the average horizontal dilution of precision (HDOP) was 0.8. These parameters indicate good quality signal acquisition [64]. In addition, to avoid interunit error, each player was assigned with the same GPS device for each training session. The reliability and validity of the 10 Hz devices were previously reported [23]. All data was acquired through a dedicated software (LaGalaColli V: 8.6.4.3) during the entire season 2019–2020.

Soccer players were categorized into six groups according to their role as follows: central back (CB), external strikers (ES), fullback (FB), midfielder (MD), striker (S), and wide midfielder (WM).

(EL indicators) considered for this study were both metabolic and neuromuscular. These are detailed in Table 1.

**Table 1:** External Load indicators descriptions

External load indicators: metabolic indicators	
*Metabolic Power*	Metabolic Power (W · kg^−1^) was calculated by multiplying EC (in J · kg^−1^ · m^−1^) by running speed (v; in m · s^−1^) at any given moment (i.e., every 0.2 s): P met = EC · v. In order to assess metabolic power, considering the energy expenditure and derived, the equation developed by di Prampero et al. [67] established on previously studies by Minetti et al. [74] and Osgnach et al. [9] was adopted
*Percentage of metabolic power*	Percentage of metabolic power with intensity > 35 W* (*%W > 35W*)*
*Number of intense actions per minute (*n° int/min)	*Number of intense actions per minute (*n° int/min) The threshold considered to establish the intense actions was 20 W · kg^−1^ which represents the metabolic power when running at a constant speed of approximately 14.4 km · h^−1^ (high speed) on grass [9]
*Distance per minute (dist/min)*	Meters covered in a minute (m/min)
*Passive Recovery time per minute*	Passive Recovery time per minute (PrT/m) represents the sum of the seconds in a minute (for each minute) in which the player moves at a metabolic power between 0 and 5 W (i.e., 0–8 km/h)
External load indicators: neuromuscular indicators	
*Percentage of intense accelerations (%int acc)*	This indicator represents the percentage of the number of accelerations > 2 m/s^2^ out of the total number of accelerations
*Percentage of intense decelerations (%int dec)*	In an opposite way to the previous one, this External load indicators indicates the percentage of the number of decelerations < -2 m/s^2^ out of the total number of decelerations
*Change of directions per min* > *30° (CdD/min* > *30)*	This External load indicators measure the number of change of directions higher than 30° per minute

### Soccer-specific tasks

The soccer-specific tasks analysed in this study are SSG and MBE. To interrupt the continuation of the play during this tasks, many soccer balls were positioned close to the playing area and no time interruptions for injuries or tactical explanations by the coach were permitted. In details:

- *Small-Sided Games (SSG)*—SSGs represent games performed in a small area of the pitch with a reduced number of players compared to regular matches and carried out with specific rules. In this study, we included different typologies of SSG based on m2/player (i.e., ~ 100 m2/player), as shown in Table 2.
- *Match-Based Exercises (MBE)*—MBEs represent soccer-specific tasks carried out in a larger area of the pitch and a relatively larger number of players compared to SSGs. Coaches determine specific rules related to tactical behaviours related similarly to performance match demand, particularly in tactical requirements. In this study, we included different typologies of MBE based on m2/player (i.e., ~ 200 m2/player), as shown in Table 3.

**Table 2:** Pitch size, Player’s area, Players number, m^2^/player, in SSGs

Pitch size (m)	Player’s area (m^2^)	Players number	m^2^/player
30 × 27	810	6vs6 + j	62
30 × 27	810	5vs5	81
36 × 30	1080	6vs6 + j	83
40 × 30	1200	7vs7	85,7
35 × 25	875	5vs5	87
30 × 30	900	5vs5	90
32 × 30	960	5vs5	96
40 × 30	1200	6vs6	100
50 × 45	2250	(7 + 7) vs7 + j	102
36 × 30	1080	5vs5	108
40 × 34	1360	6vs6	113
40 × 40	1600	7vs7	114
40 × 38	1520	6vs6 + j	117
46 × 40	1840	6vs6	120
45 × 35	1575	6vs6 + j	121
50 × 32	1600	6vs6 + j	123
40 × 36	1440	5vs5 + j	130
40 × 40	1600	6vs6	133
40 × 35	1400	5vs5	136
50 × 45	2250	7vs7 + 2j	140
40 × 32	1280	4vs4 + j	142
40 × 36	1440	5vs5	144
40 × 40	1600	5vs5 + j	145
52 × 28	1456	5vs5	145
40 × 34	1360	4vs4 + j	150
40 × 40	1600	5vs5	160
42 × 40	1680	5vs5	168
44 × 40	1760	5vs5	176

Legend. SSG, small sided games; m^2^/player, pitch square meters (m^2^)/players ‘number (calculated multiply the length of the space in metres (m) by the width of the space in metres (m) divided total players number)

**Table 3:** Pitch size, Player’s area, Players number, m^2^/player, in MBE

Pitch size (m)	Player’s area (m^2^)	Players number	m^2^/player
65 × 52	3380	10vs9	178
60 × 60	3600	10vs10	180
62 × 50	3100	8vs8 + j	182
65 × 57	3705	10vs10	185
62 × 60	3720	10vs10	186
65 × 52	3380	9vs9	188
70 × 50	3500	9vs9	194
65 × 60	3900	10vs10	195
60 × 60	3600	9vs9	200
65 × 52	3380	8vs8	211
65 × 60	3900	9vs9	216
65 × 62	4030	9vs9	224
70 × 65	4550	10vs10	227
75 × 65	4875	10vs10	244
77 × 65	5005	10vs10	250
70 × 65	4550	9vs9	253
80 × 65	5200	10vs10	260

Legend. MBE, match based exercises; m^2^/player, pitch square meters (m^2^)/ players ‘number (calculated multiply the length of the space in metres (m) by the width of the space in metres (m) divided total players number)

### Statistical analysis

Normality distribution was calculated through the Kolmogorov–Smirnov test, which revealed that all EL indicators for each task and player position was normally distributed. Means and standard deviations of both metabolic and neuromuscular EL indicators for all four tasks (i.e., OM, FM, SSG, and MBE) and for each player position (CB, ES, FB, MD, S, WM) were provided as descriptive statistics.

Two-way Analysis of Variance tests (ANOVAs) for repeated measures on one factor (training task) was performed to detect differences for each EL indicators. Tukey post-hoc test was used for pairwise comparisons for each EL indicator between both tasks and playing positions. ANOVA Effect Size (ES) was evaluated as Partial eta squared (η2p) and was used to determine the magnitude of differences classified (small effect: 0.10, medium effect: 0.30, and large effect: 0.50). The sample size power was calculated by the G*Power software (Version 3.1.9.4) using the F test family (ANOVA: Fixed Effects, special, main effects and interactions) with role (df = 5) ˟ task (conditions = 4). The sample size estimated a required sample of 149 observations required (effect size f = 0.30; *P* = 0.05; 1-β = 0.80). The Statistical Package Jamovi (The jamovi project—*jamovi* Version 1.8.0.1) was used to perform the data analysis. Graphs were created through Graph Pad Prism 8 (Version 8.0.2).

## Results

A total of 1932 individual observations among the twenty-eight players were recorded (CB = 7, ES = 7, FB = 2, MD = 5, S = 4, and WM = 3). Specifically, 387 individual observations during OM; 231 during FM; 720 during SSGs; 594 during MBEs. If categorized for role these were 338 for CB; 492 for ES; 234 for FB; 316 for MD; 126 for S and 426 for WM. Regarding the soccer-specific tasks, we collected individual data from twenty-six OM during the in-season period (i.e., ~ 300 m^2^/player), twenty FM during both pre-season and in-season periods (i.e., ~ 300 m^2^/player), twenty-eight different typologies of SSG (120.8 ± 28 m^2^/player), and twenty-six different MBE (210 ± 28 m^2^/player). Descriptive statistics of metabolic EL indicators (i.e., W, %W > 35W, n° actions int/min, dist/min, PrT/m), and neuromuscular EL indicators (i.e., % acc int, % dec int, *CdD/min* > *30*) for all four tasks (i.e., MBE, SSG, OM, and FM) and for each playing position (i.e., CB, ES, FB, MD, S, WM) are reported in Tables 4 and 5.

**Table 4:** Metabolic and intensity indicators in FM, OM SSG and MBE in different soccer playing position (Central Back, External Striker, Full Back, Midfielder, Striker, Wide Midfielder)

	MBE	SSG	OM	FM	ES (η^2^p)
Metabolic power (W)					
Central back	9.9 ± 1.8	10.5 ± 1.5^a^	8.9 ± 1.4^a, b^	9.9 ± 1.1^b, c^	Category = 0.03 Role = 0.07 Category × Role = 0.03
External striker	10.8 ± 2^1^	11.1 ± 1.7	10.1 ± 1.4^a, b, 1^	11.2 ± 0.9^a, c, 1^	
Full back	10.7 ± 1.5^1^	11.8 ± 1.9^a, 1, 2^	9.9 ± 0.8^a, b, 1^	11.3 ± 1.1^c, 1^	
Midfielder	11.3 ± 1.6^1^	10.8 ± 1.6^3^	10.7 ± 1.6^1, 3^	11.4 ± 0.9^1^	
Striker	9.9 ± 1.1^2, 4^	9.9 ± 1.4^2, 3, 4^	10.1 ± 0.8	10 ± 0.7^2, 3, 4^	
Wide midfielder	11.4 ± 1.4^1, 2, 5^	11.4 ± 1.9^1, 4, 5^	10.5 ± 1.2^a, b, 1^	11.6 ± 0.8^c, 1, 5^	
%W INT > 35W					
Central back	19 ± 6.4	18.7 ± 8.6	19.8 ± 4.3	18 ± 4.4	Category = 0.01 Role = 0.11 Category × Role = 0.02
External striker	23.9 ± 8.7^1^	23.3 ± 8^1^	25.2 ± 4.8^1^	25.6 ± 5.8^1^	
Full back	20.6 ± 5.9^2^	19.2 ± 7.5^2^	22.2 ± 3.1^b, 1, 2^	21 ± 3.4^2^	
Midfielder	15.5 ± 6.4^1, 2, 3^	18.8 ± 10.3^a, 2^	16.5 ± 2.9^1, 2, 3^	16.4 ± 5.6^2, 3^	
Striker	20.9 ± 8.2^4^	16.6 ± 9.4^a, 2^	22.3 ± 3.2^4^	22.7 ± 4.5^b, 1, 4^	
Wide midfielder	21.4 ± 6.2^2, 4^	20.1 ± 7.5^2^	21.2 ± 3.3^2, 4^	19.4 ± 4.3^2^	
N° act int/min					
Central back	1.9 ± 0.6	4 ± 1.2^a^	2.5 ± 0.6^a, b^	3.1 ± 0.5^a, b, c^	Category = 0.16 Role = 0.07 Category × Role = 0.05
External striker	2.4 ± 0.9^1^	4.5 ± 1.2^a,1^	2.9 ± 0.5^a, b, 1^	3.4 ± 0.6^a, b, c,1^	
Full back	2.1 ± 0.6	5 ± 1.4^a, 1, 2^	3 ± 0.5^a, b, 1^	3.6 ± 0.5^b, c, 1^	
Midfielder	1.6 ± 0.6^1, 2, 3^	4.2 ± 1.2^3^	3.7 ± 0.5^a, b, 1, 2, 3^	4 ± 0.5^1, 2, 3^	
Striker	2.1 ± 0.8^2, 3, 4^	3.6 ± 1.1^a, 2, 3, 4^	2.6 ± 0.4^b, 4^	2.6 ± 0.4^b, 1, 2, 3, 4^	
Wide midfielder	2.1 ± 0.6^1, 4, 5^	4.7 ± 1^a, 1, 4, 5^	3.2 ± 0.6^a, b, 1, 2, 4, 5^	3.9 ± 0.5^a, b, c, 1, 2, 5^	
Dist/Min					
Central back	104 ± 13.8	105 ± 12.9	95.3 ± 9.7^a, b^	104 ± 10.3^c^	Category = 0.04 Role = 0.10 Category × Role = 0.05
External striker	111 ± 15.8^1^	108 ± 14.2	108 ± 10.9^1^	116 ± 9.6^b, c, 1^	
Full back	111 ± 15.2^1^	117 ± 13.2^a, 1, 2^	104 ± 7.7^a, b, 1^	118 ± 11.6^c, 1^	
Midfielder	119 ± 11.8^1, 2, 3^	107 ± 13.9^a, 3^	115 ± 9.1^b, 1, 2, 3^	120 ± 10.6^b, 1^	
Striker	105 ± 10.4^4^	101 ± 11.5^2, 3, 4^	108 ± 8.2^1^	108 ± 6.8^2, 3^	
Wide midfielder	117 ± 13.3^1, 2, 5^	114 ± 12.1^1, 2, 4, 5^	109 ± 12.3^a, b, 1, 3, 4^	121 ± 8.5^b, c, 1, 5^	
T Rec pass/min					
Central back	14.8 ± 4.8	13.6 ± 4.2	20.9 ± 3.9^a, b^	17.6 ± 3.8^a, b, c^	Category = 0.11 Role = 0.05 Category × Role = 0.05
External striker	14.6 ± 6.4	14.4 ± 5.1	17.7 ± 4^a, b, 1^	13.5 ± 3.5^c, 1^	
Full back	14.1 ± 5.3	12.1 ± 3.5^a, 2^	19.9 ± 3.4^a, b, 2^	13.5 ± 4^c, 1^	
Midfielder	12 ± 4^1, 2^	13.8 ± 3.9^a, 3^	16.3 ± 3.0^a, b, 1, 3^	13.2 ± 3.8^c, 1^	
Striker	12 ± 4.8^1, 2^	12.5 ± 3.6	12.9 ± 2.3^1, 2, 3^	12.6 ± 4.2^1^	
Wide midfielder	12.7 ± 4.8^1, 2^	12.3 ± 3.7^2, 4^	17.7 ± 5^a, b, 1, 3, 5^	12.6 ± 3.1^c, 1^	

*P* < 0.05 for differences between playing positions within each task (1 difference with central back; 2 difference with external striker; 3 difference with fullback; 4 difference with midfielder; 5 difference with striker; 6 difference with wide midfielder)

*P* < 0.05 for differences between tasks for each playing position (a difference with MBE; b difference with SSG; c difference with OM; d difference with FM). Effect size (ES) are expressed as the partial eta squared (η^2^p)

Legend: MBE, match-based exercises, SSG, small sided games; OM, official matches; FM, friendly matches; %W INT > 35W, Percentage of metabolic power > 35w; N° AZ INT/MIN, Number of intense actions per minute; DIST/MIN, Distance per minute; T REC PASS/MIN, Passive Recovery time /min.

The descriptive data was provided as mean ± standard deviation

**Table 5:** Neuromuscular indicators in FM, OM SSG and MBE in different soccer playing position (Central Back, External Striker, Full Back, Midfielder, Striker, Wide Midfielder). The descriptive data was provided as mean ± standard deviation

	MBE	SSG	OM	FM	ES (η^2^p)
% Acc int					
Central back	9.5 ± 2.8	12.2 ± 3.2^a^	7.7 ± 1.6^a, b^	8.7 ± 2^b^	Category = 0.43 Role = 0.04 Category × Role = 0.02
External striker	11.5 ± 2.4^1^	13.9 ± 2.6^a, 1^	8.9 ± 1.3^a, b, 1^	10.6 ± 1.6^a, b, c, 1^	
Full back	10.1 ± 2.2^2^	13.9 ± 3.3^a, 1^	8.7 ± 1.5^a, b, 1^	10.1 ± 1.3^b, 1^	
Midfielder	10.2 ± 2.2^2^	12.6 ± 3.2^a, 2, 3^	8.9 ± 1.2^a, b, 1^	9.8 ± 1.1^b, 1^	
Striker	8.1 ± 1.8^1, 2, 3, 4^	10.3 ± 3^a, 1, 2, 3, 4^	7.3 ± 0.9^b, 2, 3, 4^	7.7 ± 1.4^b, 2, 3^	
Wide midfielder	10.9 ± 2.2^1, 5^	13.5 ± 3.0^a, 1, 5^	9.1 ± 1.5^a, b, 1, 5^	10.5 ± 1.6^b, c, 1, 5^	
% Dec int					
Central back	10.9 ± 3.2	15.2 ± 3.3^a^	8.4 ± 1.6^a, b^	9.3 ± 1.8^a, b^	Category = 0.43 Role = 0.04 Category × Role = 0.02
External striker	12.1 ± 2.6^1^	15.9 ± 2.8^a^	9.0 ± 1.3^a, b^	10.5 ± 1.6^a, b, c, 1^	
Full back	11.1 ± 2.3	16.8 ± 3.5^a, 1^	9.5 ± 1.6^a, b, 1^	10.7 ± 1.3^b, 1^	
Midfielder	11.6 ± 2.4	15.5 ± 3^a, 3^	9.9 ± 1.2^a, b, 1^	10.6 ± 1.2^b, 1^	
Striker	8.9 ± 1.9^1, 2, 3, 4^	13.7 ± 3.2^a, 1, 2, 3, 4^	8.6 ± 0.9^b^	8.4 ± 1.34^b, 2, 3^	
Wide midfielder	11.4 ± 2.2^5^	16.1 ± 3^a, 5^	10.0 ± 1.6^a, b, 1^	11.2 ± 1.5^b, c, 1, 5^	
Cdd/Min > 30°					
Central back	17.7 ± 3.4	23.5 ± 2.8^a^	14.6 ± 2.9^a, b^	15.8 ± 2^a, b^	Category = 0.55 Role = 0.02 Category × Role = 0.02
External striker	17.0 ± 3	22.4 ± 3^a, 1^	14.4 ± 1.7^a, b^	15.1 ± 1.4^a, b^	
Full b ack	17.3 ± 2	22.5 ± 3.4^a^	15.3 ± 2.3^a, b^	15.4 ± 1.7^a, b^	
Midfielder	17.2 ± 2.2	23.0 ± 2.5^a^	15.4 ± 2.2^a, b^	14.9 ± 1.3^a, b^	
Striker	16.2 ± 2.6^1^	22.8 ± 2.4^a^	15.2 ± 1.3^b^	14.6 ± 1.4^b^	
Wide midfielder	16.9 ± 2	21.8 ± 2.6^a, 1, 4^	14.4 ± 2.1^a, b^	15.2 ± 1.2^a, b^	

*P* < 0.05 differences between playing positions within each task (1 difference with central back; 2 difference with external striker; 3 difference with fullback; 4 difference with midfielder; 5 difference with striker; 6 difference with wide midfielder)

*P* < 0.05 differences between tasks for each playing position (a difference with MBE; b difference with SSG; c difference with OM; d difference with FM). Effect size (ES) are expressed as the partial eta squared (η^2^p)

Legend: MBE, match-based exercises, SSG, small sided games; OM, official matches; FM, friendly matches; %INT ACC, Percentage of intense accelerations); %INT DEC, Percentage of intense decelerations; CdD/min > 30°, Change of direction per min > 30°.

**Fig. 1:**
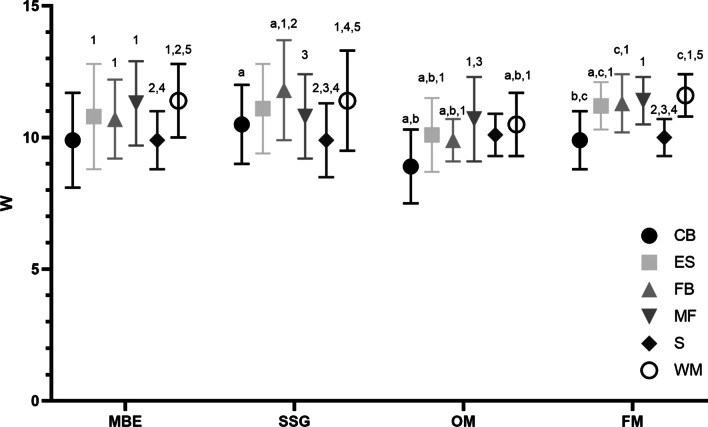
Metabolic power (W) for each role and for each task. Legend: MBE, match-based exercises, SSG, small sided games; OM, official matches; FM, friendly matches; W, Metabolic Power; CB, central back; ES, external strikers; FB, fullback;, MD, midfielder; S, striker; WM, wide midfielder. *P* < 0.05 differences between playing positions within each task (1 difference with central back; 2 difference with external striker; 3 difference with fullback; 4 difference with midfielder; 5 difference with striker; 6 difference with wide midfielder). *P* < 0.05 differences between tasks for each playing position (a difference with MBE; b difference with SSG; c difference with OM; d difference with FM)

**Fig. 2:**
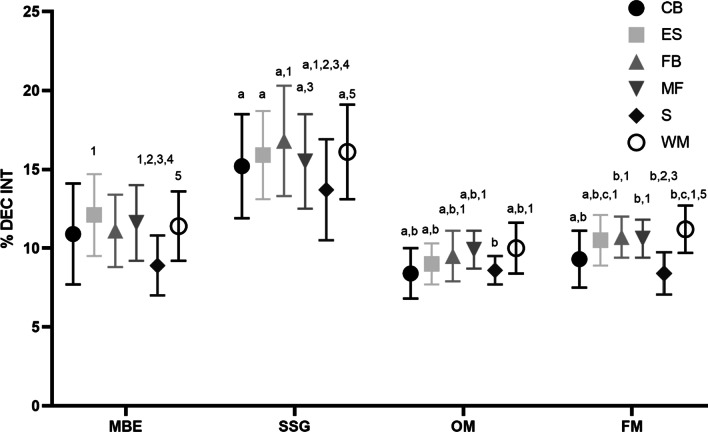
Percentage of intense deceleration (% int dec) for each role and for each task. Legend: MBE, match-based exercises, SSG, small sided games; OM, official matches; FM, friendly matches; %,DEC INT, percentage of intense deceleration; CB, central back; ES, external strikers; FB, fullback;, MD, midfielder; S, striker; WM, wide midfielder. *P* < 0.05 differences between playing positions within each task (1 difference with central back; 2 difference with external striker; 3 difference with fullback; 4 difference with midfielder; 5 difference with striker; 6 difference with wide midfielder). *P* < 0.05 differences between tasks for each playing position (a difference with MBE; b difference with SSG; c difference with OM; d difference with FM)

Results from the two-way ANOVA tests showed significant interaction, but with small effect size, in all the EL indicators between playing positions for each task and within tasks (*CdD/min* > *30*: F_(15,1921)_ = 2.41, p0.002, η^2^p = 0.02; *% dec intense*: F_(15,1921)_ = 2.41, *P* < 0.002, η^2^p = 0.02; *% acc intense*: F_(15,1921)_ = 2.09, *P* < 0.001, η^2^p = 0.02; *Watt*: F_(15,1921)_ = 4.06, *P* < 0.001, η^2^p = 0.03; dist/min: F_(15,1921)_ = 7.21, *P* < 0.001, η^2^p = 0.05; %W > 35W: F_(15,1921)_ = 2.76, *P* < 0.001, η^2^p = 0.02; n° actions int/min: F_(15,1921)_ = 6.20, *P* < 0.001, η^2^p = 0.05; PrT/m: F_(15,1921)_ = 6.85, *P* < 0.001, η^2^p = 0.05). Moreover, statistically differences, but with small effect size, between playing positions was detected in each tasks and for each EL indicator (*CdD/min* > *30*: F_(5,1921)_ = 5.99, *P* < 0.001, η^2^p = 0.015; *% dec intense*: F_(5,1921)_ = 15.35, *P* < 0.001, η^2^p = 0.04; *% acc intense*: F_(5,1921)_ = 30.22, *P* < 0.001, η^2^p = 0.07; *Watt*: F_(5,1921)_ = 30.87, *P* < 0.001, η^2^p = 0.07; dist/min: F_(5,1921)_ = 41.30, *P* < 0.001, η^2^p = 0.10; %W > 35W: F_(5,1921)_ = 49.28, *P* < 0.001, η^2^p = 0.11; n° actions int/min: F_(5,1921)_ = 29.23, *P* < 0.001, η^2^p = 0.07; PrT/m: F_(5,1921)_ = 19.55, *P* < 0.001, η^2^p = 0.05). Finally, the strongest statistical differences (with large effect size) were detected between tasks for each EL indicators (*CdD/min* > *30*: F_(3,1921)_ = 774.09, *P* < 0.001, η^2^p = 0.55; *% dec intense*: F_(3,1921)_ = 485, *P* < 0.001, η^2^p = 0.04; *% acc intense*: F_(3,1921)_ = 211,97, *P* < 0.001, η^2^p = 0.25; *Watt*: F_(3,1921)_ = 22.12, *P* < 0.001, η^2^p = 0.03; dist/min: F_(3,1921)_ = 26.54, *P* < 0.001, η^2^p = 0.04; %W > 35W: F_(3,1921)_ = 3.79, *P* < 0.010, η^2^p = 0.006; n° actions int/min: F_(3,1921)_ = 123.35, *P* < 0.001, η^2^p = 0.2; PrT/m: F_(3,1921)_ = 78.13, *P* < 0.001, η^2^p = 0.1). Details of the Tukey post-hoc analysis reporting the pairwise comparisons within and between tasks with playing positions are also provided in Tables 4 and 5. The data indicate that both metabolic and neuromuscular EL indicator differs for each role and for each task. Figures 1 and 2 show W for the metabolic and % dec int for the neuromuscular EL indicators, respectively.

## Discussion

The aim of this study was to investigate differences in both metabolic and neuromuscular EL indicators, between OM, and soccer-specific tasks (i.e., SSG, MBE, and FM) in six different semi-professional soccer playing position (CB, ES, FB, MD, S, WM). The main findings of our study showed significant differences in EL indicators between playing positions within and between each task and official matches according to our hypothesis.

### Differences in playing position within each task

In our study, significant differences in playing position among physical activity tasks were detected considering both metabolic and neuromuscular EL indicators. These results corroborate the findings of several previous studies where differences in playing position were detected during both training and competition considering EL variables derived from distance, speed and accelerations [3, 50, 51] and physiological aspects (i.e., HR and derived indices) [65, 66]. External strikers during OM show the highest performance values, while central backs and midfielders present the lowest ones. Similar results were found in soccer-specific tasks. As concern %W > 35w, we found: (1) external strikers reached the highest value, while midfielders and central backs presented the lowest results during MBE and FM; (2) a significant lower %W > 35w in central back than external strikers during SSG; (3) a significantly higher value in external strikers compared to fullbacks, midfielders, striker and wide midfielders. In order to achieve higher values of %W > 35W players need to run in larger spaces that allow higher running speeds. As a matter of fact, players positioned on the wide side present the highest values.

Considering the number of intense actions per minute as indicators of intensity we found that midfielders, wide midfielders, fullbacks and external strikers reached the highest results. Conversely, central backs and strikers showed the lowest values during OM. A similar trend was found during FM and SSG, while in MBE midfielders and central backs performed the lowest number of intense actions per minute. This result is supported by the fact that midfielders perform different running intensities compared to other players. In fact, the midfielders play in very dense central spaces that limit the performance of intense actions such as high-speed running. Differently, in MBE, wide midfielders and midfielders presented higher values of metabolic power compared to the other players’ playing positions. It is worth noting that metabolic power increases because of speed or accelerations [9, 67]. In fact, we found that in MBE midfielder performed a higher percentage of intense accelerations and decelerations than central backs and strikers. Although our sample included semi-professional soccer players, these results are in line with the findings of a previous study, carried out with elite players, showing that midfielders spent most of the time in medium and high-intensity activity during a game [51]. Moreover, midfielders present shorter recovery bouts and less time spent in very low activity [68]. Similarly, midfielders perform low to moderate-intensity activity more frequently and for longer periods [69] and stand for much less time than other players [2]. These findings suggest that midfielders show a similar pattern of physical performance regardless of the players’ level. We also found that midfielders and wide midfielders performed the highest distance per minute during OM, while central backs and fullbacks showed the lowest values. This result could be explained by the low recovery time among the actions detected in midfielders. Conversely, as concerned fullbacks, we did not observe the same results during training. In fact, fullbacks presented the highest performance during SSG.. In contrast with our results, previous studies performed in professional soccer, reported higher high-intensity speed running for this playing position. Probably, in our sample, fullbacks were required to have more defensive tactical tasks during OM than offensive ones that showed a more intense effort [4, 56].

. We found that passive recovery time per minute was significantly higher in central backs than other playing position, except for fullback. In agreement with our results, the literature reported that central backs showed the longest recoveries between consecutive high-intensity efforts [70] and spent the most time in low intensity efforts [51]. Similar results were detected during FM.Conversely, during SSG highest passive recovery was found in external strikers, midfielders and fullbacks, while the lowest values was detected in fullbacks. Players make more physical effort in wide positions requiring longer recovery time during OM. Also, central backs probably need the highest recovery time since their physical demand requires short and explosive intense actions. Conversely, during SSG, players often do not play in their usual plying position and play in very small fields with specific technical-tactical rules. Therefore, fullbacks could present higher fitness levels showing short passive recovery time per minute despite they performed high-intensity activities.

Interestingly, studies that assessed the performance considering only the speed category have underestimated the amount of high-intensity activity performed by players. Indeed, when expressed as metabolic power, central midfielders showed a higher volume of high-intensity activity compared to attackers [71]. Our study confirms previous findings showing that midfielders presented higher metabolic power during OM compared to other playing positions. In detail, metabolic power was significantly lower in central backs compared to other playing position except for strikers, during OM. Also, metabolic power was significantly lower in fullbacks compared to midfielders. Likewise, during FM metabolic power was higher for wide midfielders and midfielders than central backs and strikers. Conversely, fullbacks and wide midfielders showed the highest metabolic power while central backs and midfielders presented the lowest one during SSG.

Forwards showed the longest recovery bouts and fewer high-intensity bouts [68]. Forwards may need to recover more among the intense actions that are useful to attack the defenders of the opposite teams with unpredictability. Gaudino et al., 2013 reported that attackers covered the greatest high-speed running distance during matches [71]. Conversely, we found that strikers presented the lowest passive recovery time per minute, despite presenting higher values for a percentage of metabolic power > 35W during OM and it may be related to technical-tactical factors present in our team.

In general, previous studies showed that players in wide positions accelerated significantly more than central players [3, 72]. Specifically, Oliva-Lozano et al., 2020 reported that players covered longer distances in external positions (i.e., wide midfielder and fullback) than central midfielders [5]. Therefore, Abbott et al., 2018 found the highest intensity acceleration distances in wide attackers and wide defenders producing the highest distances due to the frequent requirement of wide positions to reach high speeds [3]. Keeping in mind that our sample was not composed of elite players, we found that central backs and strikers achieved the lowest percentage of intense accelerations and decelerations during OM while wide midfielders had the highest values highlighting that physical performance related to accelerations and decelerations depend on playing positions (central vs wide). Similar results were found during FM and MBE while strikers presented the worst (i.e., lowest intensity) values during SSG. Probably, SSG requires more homogeneous neuromuscular (i.e., acceleration, deceleration and change of directions) stimuli for all playing positions. It could be due to fewer tactical demands from players, who can move more freely without a role-dependent direction of play [41]. The low neuromuscular performance of strikers could be due to technical tactical characteristic of semi-professional soccer players.

### Differences in playing position between tasks and Official Matches

Literature reported that metabolic and neuromuscular physical demands are closely related to different typologies of soccer-specific tasks according to the training methodology in soccer [35, 43, 45, 48]. It is well known that larger spaces elicit more metabolic components as indicated by metabolic power and other derived metabolic measures than smaller areas. It is probably due to the higher speed reached by players in large fields compared to smaller ones [35]. Moreover, studies reported an increase in the acute physiological load demands (heart rate, blood lactate, and RPE) as the field dimensions increase [32, 35, 73]. Hence, larger formats are more suitable for aerobic stimuli [44] compared to small ones. Generally, literature shows an increase in accelerations, decelerations, and changes of directions in smaller pitches compared to larger ones [35, 44, 45, 48]. A study reported that regardless of the game format, defenders covered the lowest total distance, low intensity running, high intensity running, and very high intensity running. In the other hand, midfielders covered the highest total distance and high intensity running [21].

Based on these knowledge, we found significantly higher metabolic power during training than OM for all playing position, except for strikers.. Additionally, considering %W > 35W, fullbacks and strikers obtained the highest results during OM compared to SSG and MBE. Players covered a greater number of intense actions per minute during SSG than in OM, MBE, and FM. Interestingly, FM presented the highest number of intense actions per minute compared to OM for all positions except for strikers. It could be possible that higher intensity reached during FM is probably due to fewer game pauses during the match that are usually present in OM (i.e., players injury, many fouls and free-kicks, substitution, time-wasting of players and tactical aspects). Moreover, distance per minute was the lowest in OM for central backs, fullbacks and wide midfielders. Differently, external strikers, midfielders, and wide midfielders covered the highest distance per minute during FM. These results suggest that FM should be considered as a type of task with a high physical load (due to both intensity and volume).

Passive recovery time per minute showed that OM presented higher results (i.e., less intensity) compared to training tasks for all playing position except for strikers which present the same trend, which wasn’t however statistically significant. These results are in line with a previous study [55], which found that greater distances per minute were covered during SSG in comparison to matches for all playing position in elite soccer [55]. Moreover, it has been shown that a greater distance was performed both in sprinting and high intensity running for all playing position during SSG, in comparison with matches [55]. Similarly, we found that distance per minute was higher during SSG compared to OM for all playing position except for midfielders and strikers. Moreover, central backs presented the lowest intensity values both in OM and training tasks. Midfielders presented the highest metabolic power and distance per minute in SSG, MBE and FM; the highest number of intense actions per minute in FM and the lowest values of passive recovery time per minute, (i.e., high intensity) in MBE. Moreover, we found that in OM lower percentage of intense accelerations and decelerations were presented for all playing position compared to training tasks, while the highest rates were presented in SSG. In a similar way, changes of directions > 30° were higher in SSG for all playing position compared to OM, MBE, and FM.

## Conclusions

In order to better understand the physical demandsduring OM and training is necessary to consider bothmetabolic and neuromuscular EL indicators. The findings of this study revealed a significant difference in EL indicators within and between tasks and OM considering playing position. In particular, both metabolic and neuromuscular EL indicators highlighted a specific physical demand for each position on the pitch. A higher intensity was detected in training tasks compared to OM for all playing position inducing the fact that some tasks are not sufficient to exceed the demands in the competition for some roles on the pitch. As a matter of fact, higher strength responses caused by the high percentage of accelerations, decelerations and changes of directions were detected in small pitch areas (i.e., SSG). Additionally, the highest intense metabolic power (i.e., > 35W) was detected in wide positions.

It is worth noting that playing position along with other aspects influence high-intensity match physical performance. The different physical responses in playing position within each task could depend on the specificity of the role while each task typology elicits different responses based on the technical-tactical nature of the game. Our results agree with the literature in which professional or elite soccer players were assessed. This pattern suggests that players’ physical demand is strictly related to playing positions regardless of category level. However, the main difference compared to several previous studies concern fullback in which we found lower physical performance and this it could be related to technical-tactical requirements observed in our sample.

## Practical applications

Our findings suggest that the use of GPS can assist practitioners in designing training programs aimed to increase physical performance and possibly decreasing the risk of injury through more appropriate load management. In order to improve neuromuscular performance (i.e., accelerations, decelerations, and change of directions) coaches should propose specific tasks carried out in smaller pitches (i.e., SSG, ~ 100 m^2^/player). High-intensity stimuli represent the goal of the training process, therefore we recommend the use of MBE, SSG, and FM based on the training periodization.

We found a different physical request based on pitch size considering density (i.e., area per player, m^2^/player), which probably represents along with the specific playing position, the main factor characterizing physical request affecting neuromuscular performance and intensity. The assessment of physical demand should include metabolic power and derived measures which appear to be more appropriate than speed alone to monitor and assess the physical demands of each task and playing position, underlining the fundamental role of accelerations in soccer. Therefore, the exclusive application of speed-derived indicators to monitor intermittent activities should be limited. Finally, in order to monitor intensity among soccer-specific tasks and official matches, we recommend considering other EL indicators recorded by GPS devices along with internal load (e.g., RPE scale). Hence, practitioners should consider the different physical responses to different physical tasks and playing position to schedule the most appropriate training program.

## Data Availability

The datasets generated during and/or analysed during the current study are available from the corresponding author on reasonable request.
